# Research on Association of the Diameter of the Internal Carotid Artery Siphon and Nonarteritic Anterior Ischaemic Optic Neuropathy

**DOI:** 10.1155/2019/7910602

**Published:** 2019-09-02

**Authors:** Zhiyong Fu, Hongyang Li, Wei Wang, Yanling Wang

**Affiliations:** Department of Ophthalmology, Beijing Friendship Hospital, Capital Medical University, Beijing 100050, China

## Abstract

**Objective:**

To investigate the association of the diameter of the internal carotid artery siphon (ICAS) and nonarteritic anterior ischaemic optic neuropathy (NAION).

**Methods:**

Thirty patients clinically diagnosed with NAION (unilateral affected) who presented to Beijing Friendship Hospital from January 2017 to October 2018 were selected. The eyes suffered from NAION were enrolled as the observation group, and the fellow healthy eyes were enrolled as the control group. The following indexes were measured: diameter of the ICAS and the ophthalmic artery (OA), intima-media thickness (IMT) of the internal carotid artery (ICA), degree of stenosis of the ICA and plaque formation, and hemodynamic parameters of the ICA and the short posterior ciliary arteries (SPCAs). All the values were compared between the two groups.

**Results:**

The diameter of the ICAS in the observation group (0.30 ± 0.07 cm) significantly narrowed compared with that of the control group (0.32 ± 0.06 cm) (*P* < 0.05), but the diameter of the OA of the two groups had no significant difference. The detection rate of carotid atherosclerosis plaque, the average blood flow velocity (Vm), and the resistance index (RI) of the ICA in the observation group (46.67%, 26.81 ± 1.78 cm/s, and 0.72 ± 0.06) had significant differences compared with those of the control group (16.67%, 28.19 ± 2.75 cm/s, and 0.70 ± 0.05) (*P* < 0.05), but the gradings of ICA stenosis and IMT between the two groups had no significant differences. The peak systolic velocity (PSV) and the end diastolic velocity (EDV) of the SPCAs in the observation group (10.72 ± 2.88 cm/s and 3.43 ± 1.01 cm/s) were significantly lower than those of the control group (13.62 ± 3.93 cm/s and 4.59 ± 1.71 cm/s) (*P* < 0.05), but the RI of the SPCAs of the two groups had no significant differences.

**Conclusion:**

The diameter of the ICAS has a close relationship with NAION.

## 1. Introduction

Nonarteritic anterior ischaemic optic neuropathy (NAION) is a most common ischaemic optic neuropathy in the middle-aged and elderly characterized by a sudden, painless decrease in vision accompanied with a visual field defect and optic disc edema [1–4]. There were evidences to prove that it was vascular insufficiency in the capillary bed of the optic disc which caused the impairment of the optic nerve in NAION [5, 6], and as we know, the hypoperfusion of the SPCAs was the major cause [7]. Previous studies have discovered a link between the pathogenesis of NAION and the IMT of the carotid arteries and carotid diseases [8, 9], but to the best of our knowledge, there are few researches on the association of NAION and the diameter of the ICAS so far. The siphon segment of the ICA is one of the most commonly affected portions in the presence of carotid atherosclerosis; its character makes the narrowing of the vessel here play a more important role in the changes of lower reaches' hemodynamics, which might affect the perfusion of the target organ directly. In fact, the ICAS was specially studied from the anatomical point of view in a previous research [10], and the fact that calcification and pathological changes occur very often in this area was also noticed, but the focus of most of those researches were on the configuration of the siphon and the hemodynamic change itself; the relationship between the ICAS and the pathogenesis of NAION was rarely studied.

In the present study, we compared the diameter of the ICAS and other relative parameters of the eyes with NAION and the fellow healthy eyes. Our aim was to investigate the association of the diameter of the ICAS and NAION.

## 2. Methods

### 2.1. Study Design and Participants

Thirty patients clinically diagnosed with NAION (unilaterally affected) who presented to Beijing Friendship Hospital from January 2017 to October 2018 were selected. The mean age at diagnosis of the 30 patients was 56.4 ± 10.1 years (ranging from 41 to 72 years), and there were 18 male and 12 female. All participants provided written informed consent. This study was approved by the local ethics committee of Beijing Friendship Hospital and was conducted in conformance with the Declaration of Helsinki (the 2013 revision), the guideline of the International Conference on Harmonization of Good Clinical Practice.

Inclusion criteria for patients with NAION were as follows: (1) conformed to the diagnostic criteria of NAION [11], (2) presence of unilateral optic disc swelling on ophthalmoscopy during the acute stage, (3) first time outbreak of NAION without previous treatment, and (4) completed all the examinations and follow-up visits.

Exclusion criteria were as follows: (1) any other ophthalmic disease affecting the optic disc, including glaucoma, optic neuritis, uveitis, retinal or choroidal diseases, and trauma and (2) any neurologic disease that may affect the optic nerve, such as multiple sclerosis, Alzheimer disease, and Parkinson disease.

No participants were taking aspirin, and they had no history of cardiovascular or cerebrovascular disease.

The eyes suffered from NAION were enrolled as the observation group, and the fellow healthy eyes were enrolled as the control group.

**Figure 1:**
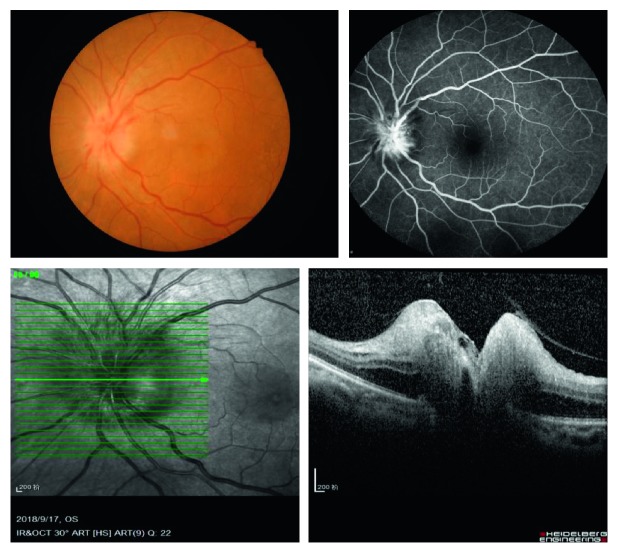
Fundus imaging. FFA and OCT showed the swollen optic disc and optic papilla surface fluorescence leakage.

Head-and-neck computed tomographic angiography (CTA) examination was performed to assess the degree of stenosis of the ICA, plaque formation, as well as the diameter of the ICAS and the OA. Color Doppler ultrasonography was performed to evaluate IMT of the ICA and the hemodynamic parameters of the ICA and SPCAs, including Vm, PSV, EDV, and RI. At the same time, fundus imaging, fundus fluorescein angiography (FFA, Heidelberg, Germany), and spectral domain optical coherence tomography scans (SD-OCT, Heidelberg, Germany) were performed (Figure 1). Finally, all the values of the two groups were compared and analyzed.

### 2.2. Examinations

#### 2.2.1. Head-and-Neck Computed Tomographic Angiography (CTA)

The examinations were performed by using the revolution CT scanner (GE, USA). Measurement of the diameter of the ICAS: firstly, choose the starting and ending points of the ICAS segment; secondly, straighten the chosen vessel using software (GE postprocessing workstation AW4.6); and finally, measure the narrowest part of the vessel defined as the diameter of the ICAS.

**Figure 2:**
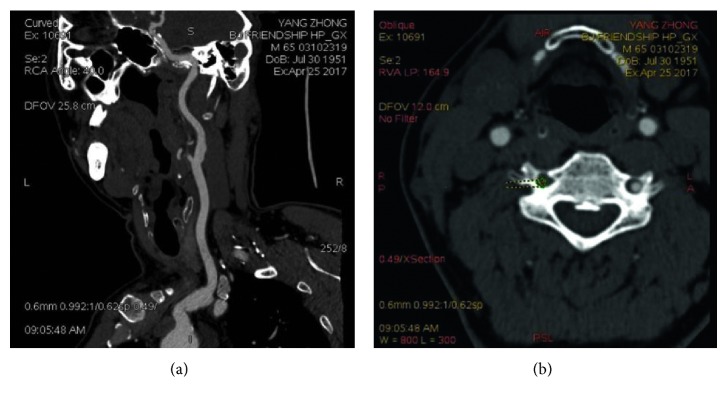
Head-and-neck CTA inspection. (a) Wall calcification of the ICAS; (b) the stenosis of the ICA.

The stenosis ratio was calculated by the NASCET (North American Symptomatic Carotid Endarterectomy Trial) stenosis grading method and was used for estimating the percentage of stenosis and classification. Vascular stenosis degree % = normal vessel diameter (*D*) − narrow vessel diameter (*N*), divided by the normal vessel diameter (*D*) × 100%. The patients with ICA stenosis were defined as third degree according to the results of the CTA examination: mild stenosis (ipsilateral carotid artery stenosis rate ≤ 29%), moderate stenosis (ipsilateral carotid artery stenosis rate of 30–69%), and severe stenosis (ipsilateral carotid artery stenosis rate of 70–100%) (Figure 2).

#### 2.2.2. Color Doppler Flow Imaging (CDFI) Examinations

**Figure 3:**
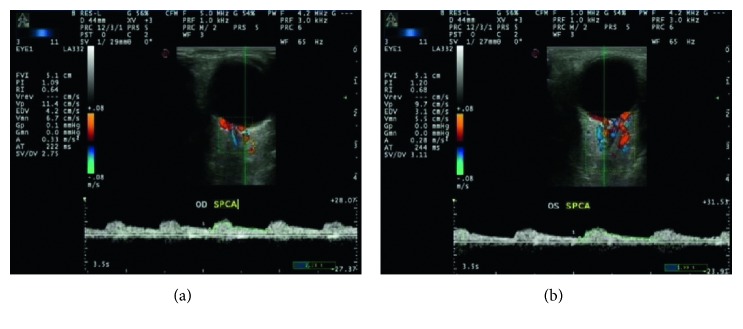
CDFI examinations of the SPCAs and the measurement process. The major parameters are PSV, EDV, and RI.

IMT and blood flow velocities of the ICA were found using a GE logiq 7 instrument (GE, USA); with the use of a linear transducer of 5–10 MHz, the main parameters such as IMT, Vm, and RI were found. Blood flow velocities of the SPCAs were found using a Esaote Mylab ClassC LA332 instrument (Esaote, Italy), with the use of a linear transducer of 3–11 MHz. The main parameters are PSV, EDV, and RI (Figure 3). All measurements were performed in the supine position.

### 2.3. Statistical Analysis

Data were expressed as mean ± SEM. The Kolmogorov–Smirnov test was used to identify the normality of distribution. Differences in the parameters between the two groups were compared using the paired *t* test or the chi-square test. A *P* value < 0.05 was considered statistically significant. Statistical analysis was performed using the IBM SPSS software version 21.

## 3. Results

### 3.1. Comparison between the Diameter of the ICAS and the OA of the Two Groups

Considering the diameter of the OA, the difference between the two groups had no statistical significance (*P*=0.899), but considering the diameter of the ICAS, the difference between the observation group (0.30 ± 0.07 cm) and the control group (0.32 ± 0.06 cm) (*t* = −2.596, *P*=0.015) had statistical significance (Table 1).

### 3.2. Comparison between the Carotid Atherosclerosis Plaque Detection Rate of the Two Groups

There were three types of carotid atherosclerosis plaque (soft, hard, and mixed plaque) found in the observation group and two types of carotid atherosclerosis plaque (soft and hard plaque) found in the control group. Considering the plaque detection rate, the difference between the observation group (46.67%) and the control group (16.67%) had statistical significance (*X*^2^ = 6.239, *P*=0.012) (Table 2).

### 3.3. Comparison between Gradings of Ipsilateral ICA Stenosis of the Two Groups

Eight eyes (26.67%) with mild ipsilateral ICA stenosis, 3 eyes (10.00%) with moderate stenosis, and 1 eye (3.33%) with severe stenosis were found in the observation group, whereas 7 eyes (23.33%) with mild stenosis and 2 eyes (6.67%) with moderate stenosis were found in the control group; the difference between the two groups had no statistical significance (*X*^2^ = 0.659, *P*=0.589) (Table 3).

### 3.4. Comparison between IMT and Hemodynamic Parameters of the ICA of the Two Groups

Considering the IMT of ICA, the difference between the two groups had no statistical significance (*t* = 1.103, *P*=0.279), but considering Vm and RI of ICA, the difference between the observation group (26.80 ± 1.79 cm/s and 0.72 ± 0.06) and the control group (28.18 ± 2.75 cm/s and 0.70 ± 0.05) had statistical significance (*t* = 3.392, *P*=0.002, and *t* = 2.988, *P*=0.006) (Table 4).

### 3.5. Comparison between Hemodynamic Parameters of the SPCAs of the Two Groups

Considering PSV and EDV of the SPCAs, the difference between the observation group (10.72 ± 2.88 cm/s and 3.43 ± 1.00 cm/s) and the control group (13.62 ± 3.94 cm/s and 4.59 ± 1.70 cm/s) had statistical significance (*t* = 4.226, *P* < 0.001, and *t* = 2.231, *P* < 0.001). Considering the RI of the SPCAs, the difference between the two groups had no statistical significance (*t* = −1.474, *P*=0.151) (Table 5).

## 4. Discussion

NAION is a major nonglaucomatous optic neuropathy. The optic nerve impairment is caused by the infarction of the laminar or retrolaminar portion of the optic nerve head supplied by the SPCAs [12, 13], which arise from the OA as it crosses the optic nerve. Patients with predisposing vascular risk factors are prone to develop such optic nerve impairment into NAION [4, 14, 15]. NAION is a multifactorial disease, and a variety of risk factors are associated with NAION, including local factors, systemic factors, and even genetic mutations [16]. Furthermore, many of these risk factors co-occur and interreact with each other. Increased values of mean platelet volume (MPV) have been reported to be a risk factor for deep venous thrombosis, acute ischaemic cerebrovascular events, acute myocardial infarction and NAION [17–22], and thrombophilia due to MPV. Hypercholesterolemia and high fibrinogen levels may contribute to the pathogenesis of NAION. On the other hand, it has been reported that glucose-6-phosphate dehydrogenase (G6PDH) deficiency has a protective effect against the development of NAION because of the reduced ability to esterify and accumulate cholesterol in the arteries in G6PDH-deficient subjects [23, 24]. The present study was an analysis of imaging features. No blood samples were taken at the time of diagnosis of NAION; thus, we could not discuss any item of hemorheology here. In order to exclude potential mixed factors which may influence the results, we designed a self-control study. Every pair of relative parameters was acquired from the same individual and compared with that of the affected eyes of patients with unilateral NAION and the unaffected healthy eyes. By means of such design, we hoped the conclusion could be more convincing.

The ICAS is always an enigmatic region implicated in cephalic and ocular ischaemic diseases. In previous studies, the ICA was divided into seven segments according to Bouthillier's classification [25]: cervical segment, petrous segment, lacerum segment, cavernous segment, clinoidal segment, ophthalmic segment, and communicating segment. The tortuous, S-shaped course (including the cavernous and clinoidal segments) has been defined as the siphon. Blood flow and morphology of the vessel are closely related: the vascular bending shape would have effects on the shear force of the vessel wall and can cause the blood flow turbulence. The tortuous configuration of the ICAS is crucial for the change of blood flow parameters, which influences circulatory dynamics of blood supply; the bending shape of the ICAS is also very important for diseases related to turbulent circulation [18, 26]. Therefore, we assumed that there was some link between the vessel diameter of the ICAS and NAION. We wanted to verify this association by means of observing the sequential hemodynamic changes, with the intention of exploring the clinical significance of the ICAS.

In our study, we found that the diameter of the ICAS distinctly narrowed in the observation group; in other words, the patients suffering from NAION showed stenosis in ipsilateral ICAS of affected eyes. Also, we found that RI of the ICA enhanced because of the narrowing of the ICAS, and Vm of the ICA decreased accordingly. The lowering of velocity due to bending in the ICAS definitely diminishes the volume of blood supply to the lower reaches region. However, the degree of ICA stenosis had no significant differences; this finding was in accordance with previous reports [19–21, 27–29]. From above, we noticed that it was the siphon but not other segments of the ICA which played a more important role in the occurrence of NAION. On the other hand, the detection rate of carotid atherosclerosis plaque in the observation group was more than that in the control group, because the low shear force on the vessel wall and the turbulence caused by variant configuration of the ICAS would induce the formation of the atherosclerotic plaque and calcification. Atherosclerotic plaque could cause the narrowing of the blood vessel, which has influence on blood flow, turbulent flow, and low shear force area in the lower reaches of plaque. We could find that this is a vicious circle; therefore, atherosclerotic plaque was a result as well as a reason. An increase in the IMT was regarded as a beginning of atherosclerosis, but we found no significant differences between the two groups in the present study. The reason may be a relatively small number of patients, which indicates that the fellow healthy eyes should be kept on follow-up.

Furthermore, the changes in hemodynamic parameters of the SPCAs were also detected. Previous research showed considerably reduced blood flow velocities in the nasal PCA of patients with NAION compared with age-matched healthy controls [30], and another previously published study that evaluated the retrobulbar hemodynamics in NAION before and after optic nerve sheath decompression also showed preoperatively lower PSVs in the mean PCAs compared with the contralateral eye [31]. However, Hayreh [32] pointed out that CDFI in the SPCAs are not relevant to the optic nerve head circulation in practice, for it is simply impossible for CDFI to differentiate the individual paraoptic branches of the SPCAs. Actually, the real question is the resolution of CDFI is insufficient till now to provide accurate measurements while viewing the vessel size of the SPCAs. Considering that the blood flow through the paraoptic branches is a fraction of the flow through the PCAs, and all the SPCAs are lying jumbled and intertwined as a vascular bundle, we determined that we could obtain an “averaged” result about the optic nerve circulation. By numerous tests performed on the eyes of our team members, we tried to find a more appropriate position and angle to gain information. In order to make the data more reliable, all measurements were performed three times by one experienced operator. We found that when the RI of the ICA enhanced and the Vm of the ICA decreased, the secondary effect was that the PSV and EDV of the SPCAs decreased significantly and RI of the SPCAs increased distinctly. PSV reflects blood vessel filling and blood supply strength, EDV reflects the blood perfusion situation of the distal tissue, and an increased RI shows high resistance in the distal vascular bed and less blood flow. All the hemodynamic changes caused hypoperfusion of the optic disc. Ischaemia might lead to progressive nerve damage and contribute to the development of NAION eventually.

There are several limitations in our study, including a relatively small number of patients with NAION and the lack of direct evidence to prove the association of the ICAS stenosis and NAION. Thus, larger sample size and animal models are necessary for future research.

Another limitation of our study is that what we measured was flow velocity and not flow volume. However, the relation of flow velocity to flow volume in the vascular bed still remains unclear. As we know, blood is a non-Newtonian fluid, the perfusion pressure, vessel caliber, plasma viscosity, or the elasticity of the vascular wall could have influence on blood flow. Because of lack of hemorheology analysis, the results are hard to completely avoid bias. But at any rate, since all parameters were acquired from the same patients with unilateral NAION and compared with their uninvolved side, the results might partly reflect the hemodynamic status at the acute stage of NAION. An adequately controlled study about flow volume and hemorheology analysis would be worthy of further workup.

Another potential limitation is that we could not exclude all mixed factors; for example, intermittent use of phosphodiesterase type 5 inhibitors (PDE5i) was reported to be associated with acute NAION onset [33]. Fortunately, PDE5i is rarely used in the elderly in China.

## 5. Conclusions

We concluded that in patients with NAION, the narrowing of the diameter of the ICAS resulted in the reduction of optic disc perfusion by hemodynamic change of the SPCAs, and the diameter of the ICAS has a close relationship with NAION.

## Figures and Tables

**Table 1:** The diameter of the ICA siphon and the OA.

	OA diameter (cm)	ICAS diameter (cm)
Observation group	0.16 ± 0.02	0.30 ± 0.07
Control group	0.15 ± 0.02	0.32 ± 0.06

*t* value	0.128	−2.596
*P* value	0.899	0.015

OA: ophthalmic artery; ICAS: internal carotid artery siphon. *P* values were calculated using the paired *t*-test.

**Table 2:** Comparison between carotid atherosclerosis plaque of the two groups.

	Soft plaque (*n*)	Hard plaque (*n*)	Mixed plaque (*n*)	Plaque detection rate (%)
Observation group	7	5	2	46.67
Control group	3	2	0	16.67

*X* ^2^ value	6.239			
*P* value	0.012			

*P* values were calculated using the chi-square test.

**Table 3:** Different gradings of ipsilateral ICA stenosis.

	Mild stenosis, *n* (%)	Moderate stenosis, *n* (%)	Severe stenosis, *n* (%)
Observation group	8 (26.67%)	3 (10.00%)	1 (3.33%)
Control group	7 (23.33%)	2 (6.67%)	0

*X* ^2^ value	0.659		
*P* value	0.589		

ICA: internal carotid artery. *P* values were calculated using the chi-square test.

**Table 4:** IMT and hemodynamic parameters of the ICA.

	IMT (mm)	Vm (cm/s)	RI
Observation group	0.73 ± 0.05	26.81 ± 1.78	0.72 ± 0.06
Control group	0.73 ± 0.04	28.19 ± 2.75	0.70 ± 0.05

*t* value	1.103	3.385	2.988
*P* value	0.279	0.002	0.006

ICA: internal carotid artery; IMT: intima-media thickness; Vm: average blood flow velocity; RI: resistance index. *P* values were calculated using the paired *t*-test.

**Table 5:** Hemodynamic parameters of the SPCAs.

	PSV (cm/s)	EDV (cm/s)	RI
Observation group	10.72 ± 2.88	3.43 ± 1.01	0.68 ± 0.06
Control group	13.62 ± 3.93	4.59 ± 1.71	0.65 ± 0.07

*t* value	4.221	4.223	−1.474
*P* Value	<0.001	<0.001	0.151

SPCAs: short posterior ciliary arteries; PSV :  peak systolic velocity; EDV: end diastolic velocity; RI: resistance index. *P* values were calculated using the paired *t*-test.

## Data Availability

The data used to support the findings of this study are included in the Supplementary Materials.

## Supplementary Materials

Supplementary Materials ICAS D (affected) cm: the diameter of the internal carotid artery siphon in the observation group (centimeter); OA D (affected) cm: the diameter of the ophthalmic artery (NAION eye) (centimeter); ICAS D (health) cm: the diameter of the internal carotid artery siphon in the control group (centimeter); OA D (health) cm: the diameter of the ophthalmic artery (the fellow healthy eyes) (centimeter); group: 0 = observation group 1 = control group; plaque: carotid atherosclerosis plaque; plaque(modified): modified carotid atherosclerosis plaque number according to statistical requirements; stenosis: ipsilateral internal carotid artery stenosis; stenosis(modified): modified ipsilateral internal carotid artery stenosis according to statistical requirements; IMT (affected): intima-media thickness of the internal carotid artery in the observation group; IMT (health): intima-media thickness of the internal carotid artery in the control group; Vm (health): the average blood flow velocity of the internal carotid artery in the control group; Vm (affected): the average blood flow velocity of the internal carotid artery in the observation group; RI (affected): the resistance index of the internal carotid artery in the observation group; RI (health): the resistance index of the internal carotid artery in the control group; SPCAs (health) PSV: the peak systolic velocity of the short posterior ciliary arteries in the control group; SPCAs (health) EDV: the end diastolic velocity of the short posterior ciliary arteries in the control group; SPCAs (health) RI: the resistance index of the short posterior ciliary arteries in the control group; SPCAs (affected) PSV: the peak systolic velocity of the short posterior ciliary arteries in the observation group; SPCAs (affected) EDV: the end diastolic velocity of the short posterior ciliary arteries in the observation group; SPCAs (affected) RI: the resistance index of the short posterior ciliary arteries in the observation group.
